# Proanthocyanidin and mitoglitazone suppress lipogenesis by targeting ferroptosis in metabolic dysfunction-associated steatohepatitis

**DOI:** 10.1007/s00210-025-04271-z

**Published:** 2025-05-19

**Authors:** Sohair M. Abd El-Naby, Naglaa F. Khedr, Nahla E. El-Ashmawy, Amera O. Ibrahim

**Affiliations:** 1 Biochemistry Department, Faculty of Pharmacy, Medical Campus, Tanta University, Tanta, Postal Code: 31527 Egypt; 2 Department of Pharmacology and Biochemistry, Faculty of Pharmacy, The British University in Egypt, El Sherouk, Postal Code: 11837 Egypt

**Keywords:** Ferroptosis, Glutathione peroxidase X4, Mitoglitazone, Metabolic dysfunction-associated steatohepatitis, Proanthocyanidin

## Abstract

Metabolic dysfunction-associated steatohepatitis (MASH) can progress to liver cirrhosis, increasing mortality risk. The study investigates the role of ferroptosis—an inflammatory cell death mechanism—in MASH and evaluates the therapeutic effects of mitoglitazone and proanthocyanidin in targeting ferroptosis to mitigate MASH progression. Forty male albino mice were divided into five groups (*n* = 8): normal control (NC) fed a standard chow diet and given 2% DMSO; MASH group was maintained on MASH protocol (high fructose-high fat diet); mitoglitazone (Mito) group was kept on MASH protocol and given Mito (10 mg/kg/day); proanthocyanidin (Pro) group was kept on MASH protocol and given Pro (150 mg/kg/day); Mito + Pro co-treated group was given Mito and Pro parallel with MASH protocol, all treatments for 12 weeks. MASH induction significantly (*p* < 0.001) increased liver weight, liver index, serum liver enzymes (ALT & AST), serum glucose, insulin, insulin resistance (HOMA-IR), lipid profile (total cholesterol, triglycerides, LDL-C), ferroptosis biomarkers (total iron, soluble transferrin receptor-1 (sTfR1), and expression of liver acyl-CoA synthetase long-chain family member 4 (ACSL4) with diffused macrovesicular severe steatosis, and inflammatory cells infiltration in liver tissues compared to NC. However, HDL-cholesterol, ferroptosis biomarkers (liver glutathione peroxidase X4 (GPX4), and total glutathione peroxidase (GPX) activities and glutathione (GSH) content) were reduced significantly (*p* < 0.001) in MASH group compared to NC. On the other hand, Mito, Pro, and their combination significantly improved ferroptotic biomarkers (GSH, GPX4, sTFR1, and total iron and ACSL-4 gene expression), glucose homeostasis, lipid profile, liver enzymes, and histology compared to MASH group. Combining the insulin-sensitizing properties with targeting of ferroptosis, by the co-treatment with mitoglitazone (MSDC-0160) and proanthocyanidin, could be beneficial in inhibition of lipogenesis with retardation of MASH development in mice.

**Graphical Abstract:**

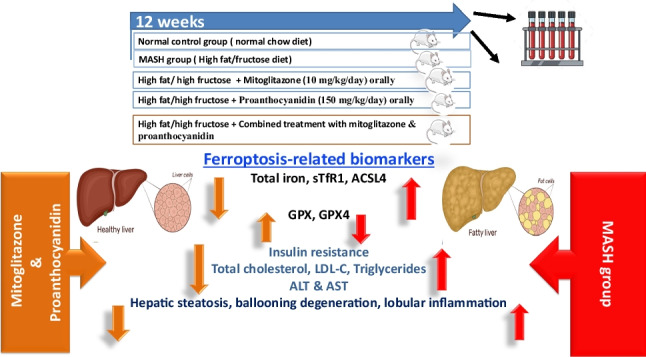

## Introduction

Metabolic dysfunction-associated steatotic liver disease (MASLD), formerly known as nonalcoholic fatty liver disease (NAFLD) is one of the most common chronic liver diseases and becoming a major cause of liver-related morbidity worldwide, impacting nearly 25% of the global population (Pouwels et al. 2022; Rinella et al. 2023). It is typically associated with obesity, insulin resistance, diabetes, and dyslipidemia. Nonalcoholic steatohepatitis (NASH) is now replaced with the term metabolic dysfunction-associated steatohepatitis (MASH). MASH is defined as a liver manifestation of a metabolic disorder and is considered the most severe form of MASLD characterized by fat accumulation and inflammation (Loomba et al. 2021).

Ferroptosis was first identified in 2012 as an iron-dependent and lipid peroxidation-driven controlled cell death. Hepatic ferroptosis plays an important role as the trigger for initiating inflammation in steatohepatitis and may provide a hopeful and attractive therapeutic target for preventing the onset and progression of MASH (Tsurusaki et al. 2019). Iron is well-known for stimulating the formation of reactive oxygen species (ROS) via Fenton’s reaction. It has previously been observed that there is a link between iron overload, disturbance of homeostasis, metabolic syndrome, and MASH (Miyanishi et al. 2019).

Fatty acids are hydrocarbon chains topped with a carboxyl group. Fatty acid metabolism is divided into the anabolic (fatty acid synthesis and lipogenesis) and catabolic (lipolysis and fatty acid oxidation) pathways, which are fine-tuned by several enzymes. Acyl-CoA synthetase long-chain family (ACSL), which is expressed on the endoplasmic reticulum and mitochondrial outer membrane, can catalyze fatty acids to form acyl-CoAs. Acyl-CoA synthetase long-chain family member 4 (ACSL4) is involved in ferroptosis mechanism. As lipid metabolic intermediates, acyl-CoAs initiate fatty acid metabolism and membrane modifications (Guo 2022).

The process of lipid peroxidation (LPO) is crucial in causing ferroptosis. Glutathione peroxidase-4 (GPX4) is an antioxidant defense enzyme. Converging information on the enzymology of GPX4 and the mechanism of LPO suggests that LPO is initiated by decomposition of hydroperoxide derivatives of lipids (LOOH) by ferrous iron leading to ferroptotic cell death. In addition, LPO has been identified as the executor of ferroptosis which leads to either GSH depletion or GPX4 inhibition (Sun et al. 2018; Ursini and Maiorino 2020).

Ferroptosis takes place when iron threshold has been exceeded. Iron transport in the plasma is carried out by transferrin. Transferrin receptor 1 (TfR1) can transfer transferrin into cells through receptor-mediated endocytosis, and release iron carried by transferrin into the iron pool. Fragment of TfR on the cell membrane is secreted into the circulation by protease hydrolysis releasing soluble transferrin receptor (sTfR), which can indirectly reflect the cytosolic expression of TfR. TfR1 has been identified as a specific target antigen related to ferroptosis (Sun et al. 2023).

Thiazolidinedione (TZD) has been shown to be efficacious in inflammatory diseases as psoriasis, ulcerative colitis, and MASH. In the case of MASH, TZD drugs improve insulin sensitivity, glucose metabolism, inflammation, and liver fibrosis (He et al. 2016). One of the recent analogs of TZD drugs is MSDC-0160 (mitoglitazone). Mitoglitazone (Mito) was found to exert significant inhibitory effects on erastin-induced ferroptosis by suppressing mitochondrial membrane potential hyperpolarization and lipid ROS production in HEK293 cells. Moreover, it has been reported that pretreatment with Mito remarkably alleviated ischemia/reperfusion (I/R) induced injury in C57BL/6 N mice by inhibiting cell death and inflammation, up-regulating GPX4, and reducing iron-related lipid peroxidation (Qi et al. 2023).

Proanthocyanidins are a class of polyphenols that possess a wide range of health benefits. Grape seed proanthocyanidin extract is a major source of proanthocyanidins (Rodríguez-Pérez et al. 2019). The physiological activities of proanthocyanidins (Pro) include reduction of oxidative stress and inflammation and alleviation of metabolic syndrome. Pro also provides multi-organ protection from various drug- and chemical-induced toxicities (Mannino et al. 2021). Moreover, previous studies suggested that proanthocyanidins can attenuate steatohepatitis *via* improving hepatic lipid metabolism and by decreasing oxidative stress and inflammation (Rauf et al. 2019). In this study, we aimed to investigate the role of ferroptotic cell death in MASH pathogenesis and progression, as well as to elucidate the underlying molecular mechanisms of mitoglitazone and proanthocyanidins either alone or in combination as ferroptotic modulators in MASH-induced mice model.

## Materials and methods

### Chemicals and drugs

Mitoglitazone (purity ≥ 98%) was purchased from MedChemExpress (USA). It was dissolved in 10% dimethyl sulfoxide (DMSO) to prepare concentration of 0.4 mg/mL. Grape seed extract proanthocyanidin (purity ≥ 96%) was obtained as a gift from Arab® Company for Pharmaceuticals and Medicinal Plants (Sharkeya, Egypt). Proanthocyanidin is dissolved in 10% DMSO to prepare concentration of 6 mg/mL. DMSO was purchased from Elgomhouria for Pharmaceuticals and Chemicals (Mansoura, Egypt).

### Experimental animals

Forty male albino Swiss mice weighing 25–30 g were bought from the National Research Center (NRC), Cairo, Egypt. For acclimatization, animals were housed for 1 week at 25 °C in plastic cages, fed regular pellet chow (El-Nasr Chemical Company, Cairo, Egypt) and given free access to water. The standard normal chow diet contained 23% protein, 48% carbohydrates, 10% fat, 17.2% fiber, 0.8% phosphorus, and 1% calcium with a total calories of 3.74 kcal/g.

### MASH protocol

For induction of MASH, mice were maintained on high fructose—high fat diet (HF/HFD) for 12 weeks (El-Ashmawy et al. 2019; Yustisia et al. 2022). The high fat diet (HFD) contained 12.2% protein, 41% carbohydrates, 40.5% fat, 4.5% fiber, 0.8% phosphorus and 1% calcium with a total calories of 5.77 kcal/g. The components of HFD were mostly of high commercial grade. The diet was prepared weekly where the ingredients were mixed and stored at 25 °C to prevent oxidation and rancidity. Twenty-five percent fructose (Sigma Aldrich, USA) was added in the drinking water and given ad libitum for the whole period of the experiment.

### Animal groups

According to treatment, animals were randomly and equally (*n*=8) divided into five groups:

- Normal control (NC): mice were given normal chow diet and ad libitum tap water for 12 weeks.
- MASH group: mice maintained on HF/HFD diet and were given 10% DMSO as a vehicle *via* oral gavage daily for 12 weeks.
- Mitoglitazone group (Mito): mice were given 10 mg/kg/day mitoglitazone orally daily parallel with MASH induction protocol for 12 weeks. The dose was selected according to previous studies that showed mitoglitazone inactivates the mitochondrial pyruvate carrier (MPC; IC50 = 1.2 µM) without affecting peroxisome proliferator-activated receptor γ (PPARγ; IC50 = 31.65 µM) in vitro. Mito enhances the rate of insulin-stimulated lipogenesis in 3 T3-L1 adipocytes in a dose-dependent manner. The dietary administration of Mito (100 mg/kg) lowers blood glucose levels in obese, hyperglycemic, hyperinsulinemic, and insulin-resistant KKAγ mice (Ghosh et al. 2016; Rohatgi et al. 2013).
- Proanthocyanidin (Pro) group: mice were given 150-mg/kg/day proanthocyanidin, orally daily for 12 weeks parallel with MASH induction protocol. The dose was selected according to previous studies in various animal models that Pro has anticancer, antioxidant and antidepressent effects (Sherif et al. 2017).
- Mitoglitazone and proanthocyanidin co-treated (Mito + Pro) group: mice in this group received both treatments of mitoglitazone and proanthocyanidin orally daily for 12 weeks with the same doses parallel with MASH induction protocol. Weights of the animals of all groups were recorded weekly.

### Blood collection and tissue sampling

At the end of the experiment, mice were fasted overnight. Blood was collected from retro-orbital sinus using capillary tube under slight isoflurane anesthesia. Serum was separated by centrifugation at 3000 rpm (Sigma centrifuge 3 K15 SIGMA Laborzentrifugen GmbH- Germany) for 20 min and stored at − 20 °C until biochemical analysis. The liver was isolated immediately after exsanguination, washed in saline, and dried on filter paper. The liver was weighed, and liver index was calculated according to the following formula (El-Ashmawy et al. 2015): (liver weight (g) × 100/mouse body weight (g)). The middle lobe of liver was fixed in 10% neutral buffered formalin for histopathological examination. The remaining liver tissue was frozen at − 80 °C until biochemical and qRT-PCR analysis.

### Determination of serum ALT and AST enzymes and lipid profile

Serum ALT and AST activities were assessed colorimetrically using commercial kits (Cayman Chemical, Co., USA) according to the manufacturer’s instructions (Wilkinson et al. 1972). Serum total cholesterol, HDL-C, LDL-C, and triglycerides (TGs) were assessed colorimetrically using commercial kits (Biodiagnostic Co., Giza, Egypt) (Bucolo and David 1973; Friedewald et al. 1972).

### Determination of glucose homeostasis

Fasting blood glucose level was measured by an enzymatic colorimetric technique (Trinder 1969) using kits purchased from Biodiagnostic Co. (Giza, Egypt). Insulin level was measured using insulin enzyme linked immunoassay (ELISA) kit (Calbiotech Inc., USA) according to the manufacturer’s instructions. Insulin resistance was calculated according to homeostasis model assessment of insulin resistance (HOMA-IR) by the following formula: HOMA-IR = [fasting plasma insulin level (μIU/mL) × fasting plasma glucose level (mg/dL)]/405 (Matthews et al. 1985).

### Determination of ferroptosis related biomarkers

#### Serum total iron

Total iron concentration was measured using commercial kits (Egyptian Company for Biotechnology Spectrum Diagnostics, Egypt). Serum was deproteinized by trichloroacetic acid and iron was dissociated from the protein transferrin by hydrochloric acid, then reduced to ferrous by thioglycolic acid. The colored complex of iron was formed with bathophenanthroline and measured colorimetrically at 535 (Wootton 1958). Linearity was up to 800 μg/dL.

#### Serum soluble transferrin receptor-1 concentration

Soluble transferrin receptor-1 (sTRF-1) was determined in serum using particle enhanced immunoturbidimetric assay (Cotton et al. 2000; Pfeiffer et al. 2007). sTRF-1 agglutinates with latex particles coated with anti-soluble transferrin receptor antibodies (Tina-quant Soluble Transferrin Receptor, Roche Diagnostics, Indianapolis, USA). The precipitate was determined photometrically on a Cobas-c 501 analyzer (Roche Diagnostics, Indianapolis, USA) at 800 nm.

#### Liver glutathione content

Liver tissue was homogenized in cold buffer (50 mM potassium phosphate and 1 mM EDTA, pH 7.5) in ratio (1:5 w/v) using tissue homogenizer (Polytron homogenizer, PT 3100, Switzerland), then the homogenate was centrifuged at 3000 rpm for 15 min at 4 °C (Sigma centrifuge 3 K15 SIGMA Laborzentrifugen GmbH, Germany), and the supernatant was collected. Liver GSH was assayed in the supernatant using purchased kits (Biodiagnostic Co., Giza, Egypt). The assay principle depends on the reduction of 5,5` dithiobis-2-nitrobenzoic acid with GSH to produce a yellow chromogen, which is directly proportional to GSH concentration, and its absorbance can be measured at 405 nm (Beutler et al. 1963). Linearity was up to 120 mg/dL.

#### Liver glutathione peroxidase (GPX, E.C. 1.11. 1.9) activity and glutathione peroxidase-4 concentration

Liver tissue homogenate was prepared and centrifuged as mentioned in the method of measurement of liver GSH. The supernatant was used for assaying GPX enzyme activity photometrically using purchased kits (Biodiagnostic Co., Giza, Egypt). GPX enzyme activity was determined by measuring NADPH oxidation at 340 nm. One unit of GPX activity is equivalent to the amount of protein that oxidizes 1 mmol/L NADPH per minute. The activity of GPX is expressed as U/g tissue (Flohé and Günzler 1984). GPX4 concentration was determined in the liver supernatant using mouse GPX4 ELISA kits (SunRed®, Shanghai, China) according to the manufacturer`s instructions with a sensitivity range 0.08–15 ng/mL (Ingold et al. 2018).

#### Quantitative real-time reverse transcriptase polymerase chain reaction of ACSL4

Total RNA of liver tissue was extracted using a total RNA purification kits (Thermo Scientific GeneJET RNA Purification Kit, USA) following the manufacturer’s protocol (Boom et al. 1990). 1 µg of total RNA was used for synthesis of cDNA using Quantitect reverse transcription kits (Qiagen®, Germany) according to the manufacturer’s protocol. The generated cDNA was used directly in real-time PCR, which was performed using SensiFAST™ SYBR No-ROX kits (Bioline, Germany). The relative expression level of ACSL4 gene was determined with β-actin as an internal reference gene by denaturing at 95 °C for 15 s, annealing at 60 °C for 30 s and extending at 72 °C for 30 s for 40 cycles using the following primer sequences (Invitrogen, USA): ACSL4: forward, 5′‐CCGACCTAAGGGAGTGATGA‐3′; reverse, 5′-CCTGCAGCCATAGGTAAAGC‐3′; β-actin: forward, 5′-ACTATTGGCAACGAGCGGTT‐3′; reverse, 5′-CAGGATTCCATACCCAAGAAGGA‐3′. BLAST (available at: www.ncbi.nlm.nih.gov/blast/Blast.cgi) was utilized to compare the primer and template sequences to other known sequences to check that they were unique. The relative mRNA gene expression was calculated using the 2^−ΔΔCT^ method (Schmittgen and Livak 2008).

### Liver histopathological examination

Liver tissues were fixed in 10% neutral buffered formalin for 12 h. Each sample was then soaked in paraffin, 5-μm-thick sections were cut using a microtome (Leica RM2135, Wetzlar, Germany), and stained with hematoxylin–eosin (H&E). The sections were evaluated using an optical microscope (CX43; Olympus, Japan) to examine the inflammation and hepatocellular ballooning and MASH scoring (Kleiner et al. 2005). The liver specimens were examined by an expert pathologist who was blinded to the treatment.

Hepatocellular steatosis was evaluated on a scale of 0 to 3 (steatosis < 5% of the liver parenchyma = grade 0, 6–33% of the liver parenchyma = grade 1, 34–66% of the liver parenchyma = grade 2, and more than 66% of the liver parenchyma = grade 3. Inflammatory infiltration of the cells was scored from 0 to 3 (grade 0: no infiltration, grade1: one to two foci per 200 fields, grade 2: three to four foci per 200 fields, and grade 3 more than four foci per 200 fields). In terms of hepatocellular ballooning, the specimens were categorized into classes 0–2 (grade 0: no ballooning, grade 1: few balloon cells, and grade 2: numerous cells/prominent ballooning).

### Statistical analysis

Analysis of data was performed with GraphPad Prism software version 22 (San Diego, CA, USA). All data were presented as mean ± SD. Statistical comparison among groups was performed by ANOVA followed by the Tukey’s multiple comparison tests. The significance was set at *P* < 0.05.

## Results

### Effect of proanthocyanidin and mitoglitazone on liver index

After 12 weeks of MASH induction, MASH group exhibited significant increase (*p* < 0.001) in both liver weight and liver index compared to NC group (Table 1). However, liver weights and liver index were significantly (*p* < 0.001) decreased in Mito group (by 49.174% and 32.27%, respectively), Pro group (by 49.50% and 30.86%, respectively), and Pro + Mito co-treated group (by 53.46% and 35.66%, respectively) as compared to MASH group (Table 1).

**Table 1:** Effect of treatments on liver index and glucose homeostasis of studied groups

Parameters	NC	MASH	Mito	Pro	Mito+Pro
Final Body weight (g)	30.25 ± 1.669	42.75 ± 3.57^a^	32.38 ± 1.923^b^	31.25 ± 2.12^b^	30.95 ± 1.128^b^
Liver weight (g)	1.02 ± 0.068	3.03 ± 0.14^a^	1.54 ± 0.37^ab^	1.53 ± 0.18^ab^	1.41 ± 0.18^ab^
Liver index (%)	3.40 ± 0.15	7.088 ± 0.52 ^a^	4.80 ± 1.31^ab^	4.90 ± 0.77^ab^	4.56 ± 0.66^ab^
Blood glucose (mg/dL)	84.12 ± 6.855	143.12 ± 9.36 ^a^	124.9 ± 15.55^ab^	116.8 ± 7.67^abc^	106.8 ± 3.75 ^abcd^
Insulin (μIU/mL)	20.44 ± 4	35.99 ± 0.10^a^	26.03 ± 0.12^ab^	31.97 ± 0.21^abc^	27.83 ± 0.39^abcd^
HOMA-IR	3.97 ± 0.78	12.7 ± 0.39^a^	8.00 ± 0.07^ab^	9.21 ± 0.57^abc^	7.34 ± 0.26^abcd^

Data are expressed as mean± (SD) (*n*=8). Significance was set at *p* < 0.05, using ANOVA

^a^significant *vs*. normal control group; ^b^significant *vs*. MASH control group; ^c^significant *vs*. mitoglitazone treated group; ^d^significant *vs.* proanthocyanidin treated group

*Liver index %*, liver weight (g) × 100 / mouse body weight (g)

*HOMA-IR*, [fasting plasma insulin level (μIU/mL) × fasting plasma glucose level (mg/dL)] / 405

*NC*, Normal control group maintained on normal chow diet for 12 weeks

*MASH group*, Mice maintained on high-fructose/ high-fat diet 12 weeks

*Mito*, Mice received 10 mg/kg/day mitoglitazone orally daily for 12 weeks parallel with MASH protocol

*Pro*, Mice received 150 mg/kg/day proanthocyanidins, orally daily for 12 weeks parallel with MASH protocol

*Mito + Pro*, Mice received 10 mg/kg/day mitoglitazone plus 150 mg/kg/day proanthocyanidin, orally daily for 12 weeks parallel with MASH protocol

### Effect of proanthocyanidin and mitoglitazone on glucose homeostasis

Both fasting blood glucose and insulin levels were significantly (*p* < 0.001) increased in MASH group by 70.13% and 76.07%, respectively as compared to control group, whereas Mito, Pro, and co-treatment significantly (*p* < 0.001) decreased blood glucose (by 12.73%, 18.39%, and 25.37%, respectively) and insulin levels (by 27.67%, 11.17%, and 22.67%, respectively) compared with MASH group (Table 1).

MASH group showed significant (*p* < 0.001) increase in HOMA-IR by 219.9% as compared to control group. However, insulin resistance was significantly decreased after treatment with Mito by 37.0%, Pro by 27.48%, and co-treatment by 42.2% as compared to MASH group. Pro group showed significant decrease in HOMA-IR when compared to Mito group. Moreover, the co-treated group showed a significant decrease in fasting blood glucose, insulin and HOMA-IR when compared with mono-treatment with either Mito or Pro (Table 1). Although, blood glucose, serum insulin, and HOMA-IR were improved in the treated groups, they remained significantly higher than their corresponding values in NC group (Table 1).

### Effect of proanthocyanidin and mitoglitazone on serum enzymes and lipid profile

Serum ALT and AST levels were significantly (*p* < 0.001) increased in MASH group by 253.2% and 372.3%, respectively when compared to NC group. However, treatment with Mito, Pro, and both of them significantly (*p* < 0.001) reduced serum ALT levels by 65.77%, 67.14%, and 71.38%, respectively and AST levels by 78.83%, 75.46%, and 71.77%, respectively compared with untreated MASH group. Serum ALT was nearly normalized in the co-treated group and showed a comparable level to that in NC group (Table 2).

**Table 2:** Effect of treatments on liver enzymes and lipid profile of studied groups

Parameters	NC	MASH	Mito	Pro	Mito + Pro
ALT (U/L)	77.29 ± 2.289	273 ± 2.16^a^	93.44 ± 2.425^ab^	89.71 ± 1.799^abc^	78.14 ± 2.26^bcd^
AST (U/L)	65.93 ± 1.38	311.4 ± 2.63^a^	76.43 ± 2.22^ab^	87.91 ± 1.83^abc^	70.93 ± 3.06^abcd^
Total cholesterol (mg/dL)	84.32 ± 2.652	338.2 ± 3.305^a^	147.8 ± 1.665^ab^	154.0.9 ± 1.76^abc^	136.8 ± 2.68^abcd^
Triglycerides (mg/dL)	83.83 ± 2.49	337.8 ± 3.451^a^	173.6 ± 2.112^ab^	153.6 ± 1.559^abc^	148.2 ± 1.69^abcd^
LDL-C (mg/dL)	36.51 ± 1.414	101.7 ± 2.203^a^	83.73 ± 1.554^ab^	88.1 ± 1.783^abc^	80.47 ± 1.72^abcd^
HDL-C (mg/dL)	32.24 ± 1.60	21.6 ± 2.06^a^	27.27 ± 1.721^ab^	26.2 ± 1.175^ab^	28.21 ± 1.499^ab^

Data are expressed as mean ± (SD) (*n* = 8). Significance was set at *p* < 0.05, ^a^significant vs. normal control group, ^b^significant vs. MASH control group, ^c^significant vs. mitoglitazone treated group, ^d^significant vs. proanthocyanidin treated group. *ALT* Alanine transaminase, *AST* aspartate transaminase, *LDL-C* low density lipoprotein-cholesterol, *HDL-C* high density lipoprotein-cholesterol

*NC* Normal control group maintained on normal chow diet for 12 weeks

*MASH group* Mice maintained on high-fructose/high-fat diet 12 weeks

*Mito* Mice received 10 mg/kg/day mitoglitazone orally daily for 12 weeks parallel with MASH protocol

*Pro* Mice received 150 mg/kg/day proanthocyanidins, orally daily for 12 weeks parallel with MASH protocol

*Mito* + *Pro* Mice received 10 mg/kg/day mitoglitazone plus 150 mg/kg/day proanthocyanidin, orally daily for 12 weeks parallel with MASH protocol

Serum total cholesterol, triglycerides and LDL-C concentrations were significantly (*p* < 0.001) increased in MASH group by 269.9%, 303%, and 178.55%, respectively along with significant decrease in HDL-C level by 33% compared with NC (Table 2). Mito, Pro, and combined treatment significantly (*p* < 0.001) decreased serum total cholesterol (by 58.3%, 55.56%, and 59.55%, respectively), TGs (by 48.61%, 54.53%, and 56.13%, respectively) and LDL-C (by 17.66%, 13.37%, and 20.87%, respectively) compared to untreated MASH group. In addition, HDL-C levels were significantly increased in Mito group by 26.25%, Pro group by 21.296%, and co-treated group by 30.601% compared to MASH group (Table 2). Moreover, the combined treatment with Pro and Mito showed significant decrease in total cholesterol, triglyceride, and LDL-C compared with each of the mono-treated groups but remained significantly greater than their corresponding values in NC group (Table 2).

### Effect of proanthocyanidin and mitoglitazone on liver GSH, GPX, and GPX4

**Fig. 1:**
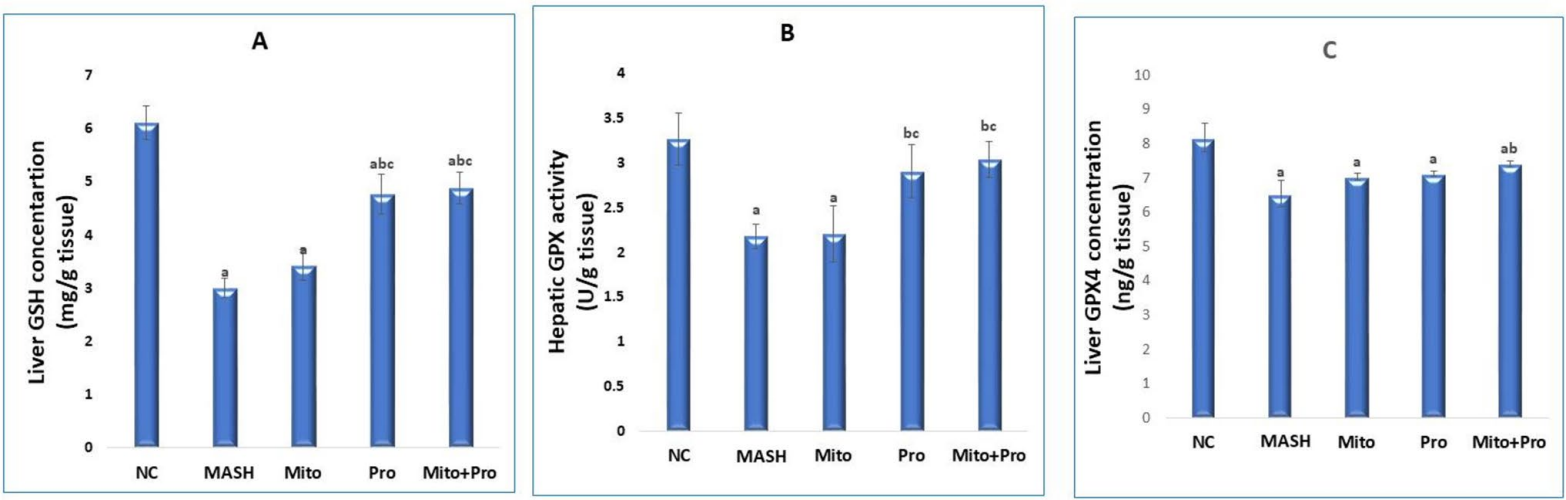
Effect of different treatments on liver GSH (**A**), GPX (**B**), and GPX4 (**C**). Data are expressed as mean ± SD (*n* = 8). Significance was set at *p* < 0.05. a: significant vs. normal control group, b: significant vs. MASH control group, c: significant vs. mitoglitazone-treated group, d: significant vs. proanthocyanidin-treated group. GSH: glutathione, GPX: glutathione peroxidase, GPX4: glutathione peroxidase-4. NC: normal control group maintained on normal chow diet for 12 weeks. MASH group: mice maintained on high-fructose/high-fat diet for 12 weeks. Mito: mice received 10 mg/kg/day mitoglitazone orally daily for 12 weeks parallel with MASH protocol. Pro: mice received 150 mg/kg/day proanthocyanidin orally daily for 12 weeks parallel with MASH protocol. Mito + Pro: mice received 10 mg/kg/day mitoglitazone plus 150 mg/kg/day proanthocyanidin, orally daily for 12 weeks parallel with MASH protocol

Liver GSH content, GPX activity and GPX4 concentration were significantly decreased in MASH group by 49.34%, 33.33%, and 20.01%, respectively compared to NC group (Fig. 1). Treatment with Pro and Mito + Pro significantly (*P* < 0.001) elevated GSH by 58.47% and 62.46%, respectively, and GPX enzyme activity by 33.49% and 39.45%, respectively, compared to MASH group (Fig. 1A and B). Liver GPX4 level was significantly (*p* < 0.0001) elevated in combined treatment with Mito + Pro compared to untreated MASH group. However, Mito and Pro mono-treated groups did not show any significant change in liver GPX4 levels when compared to MASH group (Fig. 1C).

### Effect of proanthocyanidin and mitoglitazone on serum total iron and sTfR1 levels

**Fig. 2:**
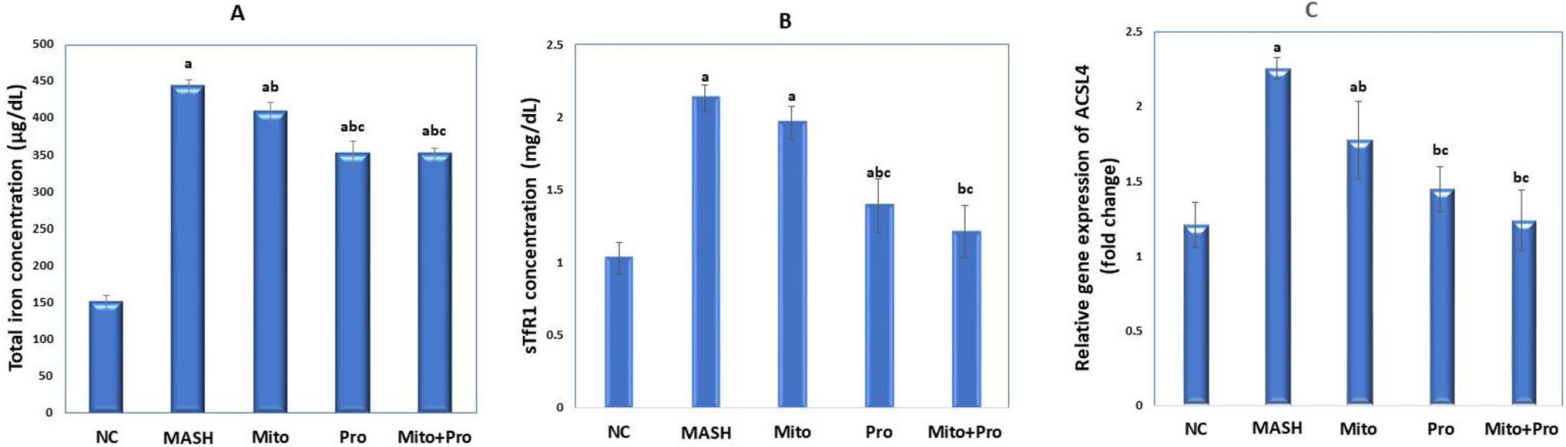
Effect of proanthocyanidin and mitoglitazone on **A** serum total iron concentration; **B** serum sTfR1 concentration; **C** relative gene expression of ACSL4. Data are expressed as mean ± SD. Significance was set at *p* < 0.05, a: significant *vs*. normal control group, b: significant vs. MASH control group, c: significant vs. mitoglitazone treated group. ACSL4: Acyl-CoA synthetase long chain family member 4, sTR1: soluble transferrin receptor-1

MASH induction was significantly (*p* < 0.0001) elevated the total iron by 194.22% and sTFR1 by 106.79%, respectively, compared to NC group. The serum total iron was significantly (*p* < 0.0001) reduced in Mito group by 7.99%, Pro group by 20.8%, and combined treatment group by 21.01% compared to untreated MASH group. In addition, Pro and combined treatment with Mito + Pro significantly (*p* < 0.0001) reduced sTFR1 by 34.74% and 43.19%, respectively compared with MASH group (Fig. 2A and B).

Both Pro group and co-treated group showed significant decrease in both total iron and sTfR1 levels when compared to Mito treated group. Single treatment with Mito did not show significant difference in sTfR1 level when compared to MASH control group, whereas, combined treatment with Pro and Mito decreased sTfR1 levels to near the normal level (Fig. 2B).

### Effect of proanthocyanidin and mitoglitazone on relative gene expression of ACSL4 in hepatic tissues

Acyl-CoA synthetase long chain family member 4 was significantly (*p*<0.001) upregulated in MASH group by 85.24% when compared to C group. However, ACSL4 relative gene expression was significantly (*p*<0.001) downregulated in Mito group by 21.23%, Pro group by 35.84%, and co-treated group by 44.69% compared to untreated MASH group (Fig. 2C). Moreover, there was no significant difference in ACSL4 gene expression between normal control group and each of Pro and co-treated groups. Pro treated group and co-treated group showed a statistically significant (*P*<0.001) downregulation in ASCL4 gene expression when compared to Mito treated group (Fig. 2C).

### Effect of proanthocyanidin and mitoglitazone on liver histopathology

**Fig. 3:**
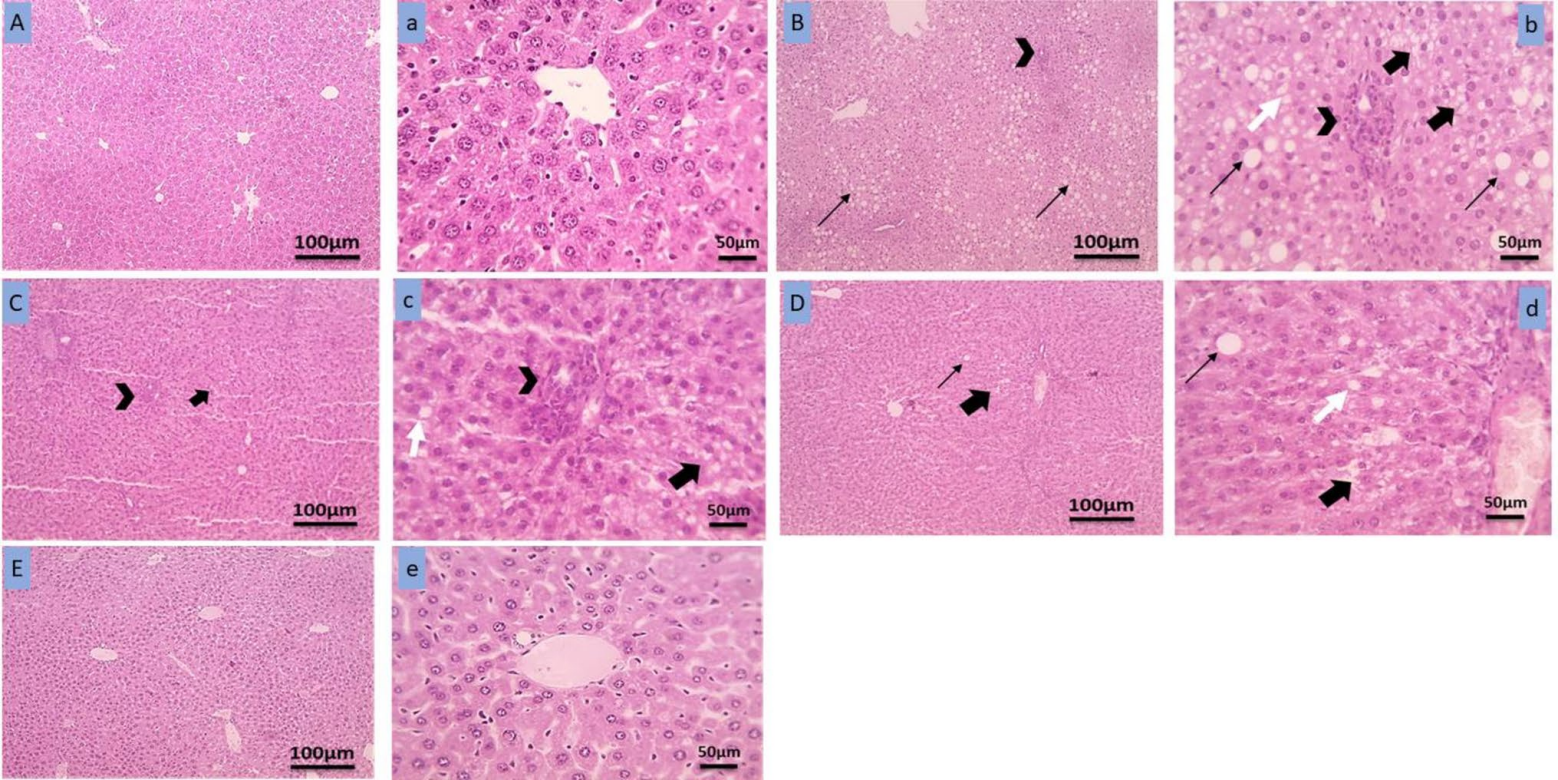
Representative photomicrograph of liver tissue sections stained with of H & E (low magnification × 100, 100 µm bar) and high magnification × 400, 50 µm bar). (**A**, a) Normal control group showing normal hepatocytes arranged in cords around the central vein. (**B**, b) MASH group showing ballooning degeneration (thick black arrow), diffuse micro- (white arrow) and macrovesicular (thin black arrow) steatosis, lobular and portal inflammation (arrowhead), narrowed to occluded sinusoids. (**C**, c) Mitoglitazone group displaying mild ballooning degeneration (thick black arrow), mild microvesicular in few hepatocytes, mild portal inflammation (arrowhead), narrowed to occluded sinusoids. (**D**, d) Proanthocyanidin group displaying focal ballooning degeneration in hepatocytes (thick black arrow), mild microvesicular steatosis (white arrow) in few hepatocytes and occasional macrovesicular steatosis (thin black arrow), re-opened sinusoids, absent portal inflammation. (**E**, e) Mitoglitazone and proanthocyanidin co-treated group displaying normal histology of hepatocytes

**Fig. 4:**
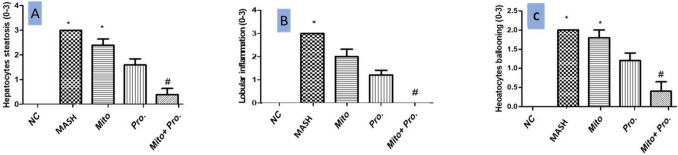
Effect of different treatments on hepatocytes steatosis (**A**), lobular inflammation (**B**), and ballooning degeneration (**C**). Bars are means ± SD. NC: normal control group maintained on normal chow diet for 12 weeks. MASH group: mice maintained on high-fructose/high-fat diet for 12 weeks. Mito: mice received 10 mg/kg/day mitoglitazone orally daily for 12 weeks parallel with MASH protocol. Pro: mice received 150 mg/kg/day proanthocyanidin, orally daily for 12 weeks parallel with MASH protocol. Mito + Pro: mice received 10 mg/kg/day mitoglitazone plus 150 mg/kg/day proanthocyanidin, orally daily for 12 weeks parallel with MASH protocol. *Significant difference from normal control group at *p* < 0.05; # significant difference from MASH group at *p* < 0.05

Results of liver histopathology are presented in Fig. 3 as photomicrographs and Fig. 4 as the statistical results of hepatic steatosis (4 A), lobular inflammation (4B), and ballooning degeneration (4 C). Liver sections from normal control group showed normal hepatocytes arranged in cords around the central vein (Fig. 3A and a). However, the liver section of MASH group (Fig. 3B, b) showed ballooning degeneration with diffuse macrovesicular severe steatosis, deep basophilic peripheral nuclei in many hepatocytes and few inflammatory cells (Fig. 4). The liver sections of Mito group (Fig. 3C and c) displayed mild ballooning degeneration, mild microvesicular in few hepatocytes, mild portal inflammation, narrowed to occluded sinusoids (Fig. 4). Liver sections of Pro group showed focal ballooning degeneration in centrilobular hepatocytes (Fig. 3D and d, Fig. 4). Liver sections of Mito and Pro co-treated group displayed normal histology of hepatocytes with mild steatosis, no lobular inflammation with little hepatocyte ballooning (Fig. 3E, e and Fig. 4). The histological improvement observed in the co-treated group was significant as compared to MASH group as shown in Fig. 4.

## Discussion

Ferroptosis is a newly identified iron-dependent atypical form of cell death that differs from apoptosis and necrosis. It involves an iron-dependent generation and accumulation of lipid peroxidation products and ROS that ultimately lead to cell death (He et al. 2023). Glutathione, lipid and iron metabolic pathways have been shown to be closely associated with the regulation of ferroptosis (Yan et al. 2021). The accumulation of iron and lipid peroxidation products in hepatocytes induces ferroptosis, which in turn aggravates MASH (Yan et al. 2021; Chen et al. 2023). Therefore**,** the present study aimed to investigate the possible protective effects of Mito and/or Pro on ferroptotic cell death in MASH mice model.

Herein, MASH group maintained on HF/HFD for 12 weeks showed significant increase in liver weight and liver index with dyslipidemia. These results were consistent with previous studies (El-Ashmawy et al. 2019; Yustisia et al. 2022) showing that the induction of MASH with HF/HFD enhanced fatty acid intake and caused excessive accumulation of fats in hepatocytes resulting in hepatocellular ballooning. The increased lipid deposition in the liver can exert prooxidative and proinflammatory effects that account for liver steatosis, injury, apoptosis, fibrosis, and necrotizing inflammation (Hamouda et al. 2022; Stål 2015). The current findings of the histopathological examination were supporting and revealed that liver tissue of MASH group exhibited ballooning degeneration with diffuse macrovesicular severe steatosis, deep basophilic peripheral nuclei in many hepatocytes, and few inflammatory cells.

Compared to the normal control, the current study demonstrated an increase in the serum enzymes ALT and AST after MASH induction. These results were in accordance with Sanyal et al., who reported that serum ALT and AST are independent indicators of MASLD progression and mortality suggesting that patients with the elevated transaminases may be eligible for ultrasonography to diagnose MASLD (Sanyal et al. 2015).

In the present study, fasting blood glucose level, serum insulin, HOMA-IR, total cholesterol, triglycerides, and LDL-C were increased significantly after MASH induction. These results were explained on the basis that hyperinsulinemia in insulin-resistant states stimulates hepatic de novo lipogenesis, which represents 25% of the hepatic triglycerides accumulating in MASLD (Smith et al. 2020), and thus, insulin resistance may contribute to the hepatic steatosis observed herein.

Despite high insulin levels, patients with hepatic steatosis during fasting have enhanced glucose synthesis due to increased hepatic gluconeogenesis. In the postprandial state, however, hepatic glucose production is suppressed by glucose-stimulated insulin secretion, and the glucose ingested is partially stored as glycogen and partially oxidized, producing pyruvate, lactate, and amino acids like alanine, which are also gluconeogenic substrates directed to de novo lipogenesis and glycerol synthesis (Gastaldelli and Cusi 2019). Excess gluconeogenic substrates (and glucose itself) can also be used to synthesize glycerol, which is then used for triglyceride synthesis. The availability of glycerol is a limiting step in hepatic triglyceride production (Gastaldelli and Cusi 2019).

In comparison with untreated MASH group, our study showed that administration of Mito and Pro, either alone or in combination, significantly reduced liver weight, liver index, liver function enzymes, blood glucose, serum insulin, and HOMA-IR as well as improved the dyslipidemia. The histopathological findings of liver tissues of treated groups showed mild ballooning degeneration, mild portal inflammation, and mild steatosis in the mono-treated groups. However, the normal hepatocyte histology was observed in the co-treated group.

Mitoglitazone is a thiazolidinedione class member that inhibits the mitochondrial pyruvate carrier with low affinity for PPARγ. The inhibition of mitochondrial pyruvate carrier leads to inhibition of gluconeogenesis from pyruvate, which in turn increases insulin sensitivity (McCommis et al. 2017). In addition, mitoglitazone decreases de novo lipogenesis by decreasing the amount of pyruvate that is metabolized in mitochondria to acetyl-CoA, an essential substrate for de novo lipogenesis (Buchanan and Taylor 2020). Thus, mitoglitazone can reduce insulin resistance, improve glucose homeostasis, reduce gluconeogenesis and prevent MASH development, which were consistent with our findings. Furthermore, McCommis et al. reported that MSDC-0602—a novel insulin sensitizer acting as a direct mitochondrial pyruvate carrier inhibitor—prevented and reversed liver fibrosis and inhibited stellate cell activation in mice livers fed a high-fat, high-fructose, high-cholesterol diet, and protected animals from developing MASH (McCommis et al. 2017).

Proanthocyanidin has been shown in previous studies to improve blood glucose by increasing glucose tissue uptake, reducing hepatic glucose production, activating AMP-activated protein kinase (AMPK) through calcium/calmodulin-dependent protein kinase kinase and increasing insulin signaling via the PI3 K/Akt pathway (Yang and Chan 2017). Such reports could explain the improvement of insulin sensitivity and glycemic index observed in our study along with the improvement of liver pathology in Pro group.

Furthermore, Liu et al. (2020) demonstrated that proanthocyanidin is considered the most effective plant antioxidant and free radical scavenger with beneficial health properties such as diabetes prevention and treatment, regulating metabolism, improving lipid metabolism, reducing fat deposition, improving insulin resistance, increasing TGs decomposition, improving brown adipose tissue heat production capacity, improving intestinal flora, lowering food intake, and improving peripheral clock to prevent further development of obesity (Liu et al. 2020).

The Fe^3+^ in the circulatory system is present in either serotransferrin-mediated or lactotransferrin-mediated iron and is combined with endocytosis through TfR1 to produce endosomes (Nie et al. 2022). In endosomes, Fe^3+^ is reduced to Fe^2+^, which is then released from the endosomes into the cytoplasm, while the excess iron is retained in ferritin. When too much Fe^2+^ iron accumulates in the cell, it accelerates the creation of ROS via Fenton reaction that stimulates lipid peroxidation and causing ferroptosis (Zhang et al. 2022). Moreover, the increased TfR1 expression, decreased ferritin expression, and decreased iron transporter expression contribute to the induction of the non-canonical ferroptosis pathway (Nie et al. 2022).

In the present study, MASH group showed significant changes in ferroptotic biomarkers indicating induction of ferroptotic cell death in MASH. There was significant decrease in liver GPX, GPX4, and GSH as well as significant increase in total serum iron and sTfR1 levels and liver ACSL4 gene expression compared with normal control group. These results were consistent with previous studies, which showed that phospholipid peroxidation substrates, bioactive iron, and ROS constitute a strong link between ferroptosis and MASH (Zhang et al. 2022).

Earlier studies demonstrated that MASLD patients experience iron overload in the liver, thereby triggering lipid peroxidation that is involved in the pathogenesis of MASH (Peleman et al. 2024). In human, sTfR1 is associated with obesity. In patients with MASLD and MASH, TfR1 expression was significantly and progressively upregulated. A cross-sectional study suggested that iron regulators are independent risk factors for hyperglycemia and iron is shown to be a vital factor in insulin sensitivity. Studies in rats showed that chronic hyperinsulinemia increases TfR1 expression by stabilizing TfR1 mRNA. In addition, 60% high fructose diet–induced obese rats accumulate hepatic iron that was associated with increased expression of TfR1 (Ameka and Hasty 2022).

GSH is an important antioxidant, and its depletion results in deactivation of GPX4, which then promotes the accumulation of lipid peroxides leading to ferroptosis initiation (Li et al. 2022). Moreover, ACSL4 is a key factor involved in regulation of ferroptosis pathway. Its overexpression promotes ferroptosis in cells because it is considered a key enzyme in lipid peroxides production (Cheng et al. 2020; Jia et al. 2023). Therefore, therapeutic targeting of ferroptosis is a promising strategy for the prevention of the onset of steatohepatitis.

In the present study, treatment with Mito significantly reduced total serum iron and ACSL4 gene expression without affecting GSH, GPX, and GPX4 compared to untreated MASH. These results were consistent with Qi et al. findings that pretreatment with Mito significantly inhibits lipid ROS production, and ferroptotic cell death in erastin-induced ferroptosis model through restoring metabolic iron balance, reducing lipid peroxidation, and protecting against mitochondrial dysfunction in renal ischemia–reperfusion injury model (Qi et al. 2023). To the best of our knowledge, this was the first study to reveal the possible treatment of MASH by mitoglitazone through targeting the ferroptosis pathway, providing new insights into mitoglitazone’s involvement in ferroptosis control.

The anti-ferroptotic property of Pro was evident in the current work, where Pro significantly increased GSH, GPX as well as significantly decreased serum total iron, sTfR1, and ACSL4 gene expression compared to MASH group. These results were in agreement with previous study where proanthocyanidin attenuated ferroptosis via decreasing ACSL4 and upregulating both GSH and GPX in acute lung injury in mice (Lv et al. 2023).

The combined treatment with both mitoglitazone and proanthocyanidin, in the current study, exhibited more pronounced and significant effects regarding glucose homeostasis, insulin resistance, and dyslipidemia, compared to each of the mono-treated groups. However, the anti-ferroptotic properties of mitoglitazone mono-treatment were less evident than that observed with proanthocyanidin mono-treatment or the combined treatment. In addition, the liver tissue from the co-treated group showed normal histology and was comparable to that of the control group, indicating hepatoprotective effect of this combination.

## Conclusion

To date, pharmacologic therapies for management of MASH have shown limited efficacy. Accumulating evidences showed that ferroptosis likely plays an essential role in MASH development. Combining the insulin sensitizing properties with targeting of ferroptosis, by the co-treatment with Mito + Pro, could be beneficial in inhibition of lipogenesis with retardation of MASH development in mice. Further studies are required to examine other potential molecular targets underlying ferroptosis inhibition by mitoglitazone and proanthocyanidin.

## Data Availability

All source data for this work (or generated in this study) are available upon reasonable request.

## Abbreviations

*MASH*: Metabolic dysfunction-associated steatohepatitis
*MASLD*: Metabolic dysfunction-associated fatty liver disease
*HF/HFD*: High-fructose/high-fat diet
*DMSO*: Dimethylsulfoxide
*Mito*: Mitoglitazone
*Pro*: Proanthocyanidin
*ALT*: Alanine aminotransferase
*AST*: Aspartate aminotransferase
*sTfR1*: Soluble transferrin receptor-1
*ACSL4*: Acyl-CoA synthetase long-chain family member 4
*GPX4*: Glutathione peroxidase-4
*GPX*: Glutathione peroxidase
*GSH*: Glutathione
*ROS*: Reactive oxygen species
*LPO*: Lipid peroxidation
*HOMA-IR*: Homeostatic model assessment for insulin resistance
*AMPK*: Adenosine monophosphate-activated protein kinase
